# Fluvoxamine inhibits Th1 and Th17 polarization and function by repressing glycolysis to attenuate autoimmune progression in type 1 diabetes

**DOI:** 10.1186/s10020-024-00791-1

**Published:** 2024-02-05

**Authors:** Yuan Zou, Jing Zhang, Fei Sun, Qianqian Xu, Longmin Chen, Xi Luo, Ting Wang, Qing Zhou, Shu Zhang, Fei Xiong, Wen Kong, Ping Yang, Qilin Yu, Shiwei Liu, Cong-Yi Wang

**Affiliations:** 1 The Center for Biomedical Research, Department of Respiratory and Critical Care Medicine, NHC Key Laboratory for Respiratory Diseases, Tongji Hospital Research Building, Tongji Hospital, Tongji Medical College, Huazhong University of Sciences and Technology, Wuhan, China; 2 Department of Rheumatology and Immunology, The Central Hospital of Wuhan, Tongji Medical College, Huazhong University of Science and Technology, Wuhan, 430000 China; 3 Department of Endocrinology, Wuhan Union Hospital, Tongji Medical College, Huazhong University of Science and Technology, Wuhan, 430000 China; 4 Department of Endocrinology, Shanxi Bethune Hospital, Shanxi Academy of Medical ScienceTongji Shanxi Hospital, The Key Laboratory of Endocrine and Metabolic Diseases of Shanxi Province, Third Hospital of Shanxi Medical University, Taiyuan, China

**Keywords:** Type 1 diabetes, Fluvoxamine, Th1 and Th17 differentiation, Glycolysis, PI3K-AKT signaling

## Abstract

**Background:**

Fluvoxamine is one of the selective serotonin reuptake inhibitors (SSRIs) that are regarded as the first-line drugs to manage mental disorders. It has been also recognized with the potential to treat inflammatory diseases and viral infection. However, the effect of fluvoxamine on autoimmune diseases, particularly type 1 diabetes (T1D) and the related cellular and molecular mechanisms, are yet to be addressed.

**Method:**

Herein in this report, we treated NOD mice with fluvoxamine for 2 weeks starting from 10-week of age to dissect the impact of fluvoxamine on the prevention of type 1 diabetes. We compared the differences of immune cells between 12-week-old control and fluvoxamine-treated mice by flow cytometry analysis. To study the mechanism involved, we extensively examined the characteristics of CD4^+^ T cells with fluvoxamine stimulation using RNA-seq analysis, real-time PCR, Western blot, and seahorse assay. Furthermore, we investigated the relevance of our data to human autoimmune diabetes.

**Result:**

Fluvoxamine not only delayed T1D onset, but also decreased T1D incidence. Moreover, fluvoxamine-treated NOD mice showed significantly attenuated insulitis coupled with well-preserved β cell function, and decreased Th1 and Th17 cells in the peripheral blood, pancreatic lymph nodes (PLNs), and spleen. Mechanistic studies revealed that fluvoxamine downregulated glycolytic process by inhibiting phosphatidylinositol 3-kinase (PI3K)-AKT signaling, by which it restrained effector T (Teff) cell differentiation and production of proinflammatory cytokines.

**Conclusion:**

Collectively, our study supports that fluvoxamine could be a viable therapeutic drug against autoimmunity in T1D setting.

**Supplementary Information:**

The online version contains supplementary material available at 10.1186/s10020-024-00791-1.

## Introduction

Type 1 diabetes (T1D) is an autoimmune disease resulting from T cell-mediated destruction of the insulin-producing β cells (Barnett 2018). Despite decades of improvement to the available insulin preparations, it remains difficult to achieve glycemic control in T1D patients, and complications resulted from poorly controlled blood glucose levels become a common event (Bluestone et al. 2021). Therefore, intervention strategies aimed at suppressing autoimmune response or promoting self-tolerance would be more rewarding to prevent disease progression and facilitate β cell recovery in comparison to the exogenous insulin therapy (von Scholten et al. 2021).

Multiple lines of evidence support that CD4^+^ T effector (Teff) cells are crucially important for priming an immune response against β cells (Boldison and Wong 2016; Haskins and Cooke 2011). CD4^+^ Teff cells not only directly secrete various proinflammatory cytokines and other soluble mediators such as nitric oxide (NO) (Jianjun et al. 2013), but also are responsible for licensing the activation of macrophages, cytotoxic CD8^+^ T cells, and B cells, which ultimately lead to the progressive insulitis along with β cell killing (Anderson and Bluestone 2005; Wållberg and Cooke 2013). CD4^+^ Teff cells comprise several distinct subsets, among which Th1 and Th17 subsets play a key role in T1D pathogenesis (Walker and von Herrath 2016). Th1 cells are regarded as a major source of type I inflammatory cytokines, including interferon IFN-γ, interleukin (IL)-1β, and tumor necrosis factor (TNF)-α, which have a profound pro-apoptotic effect on β cells (Cnop et al. 2005). Nevertheless, Th17 cells consist of a more plastic repertoire for their pathogenicity (Yasuda et al. 2019). Th17 cells induced by IL-6, IL-23, and IL-1β are generally thought to be highly pathogenic, which tend to exhibit characteristics similar as Th1 cells in the context of T1D (Haskins and Cooke 2011). Therefore, effective agents that target Th1 and Th17 cells could be potential candidate drugs for treatment of autoimmune diabetes.

Fluvoxamine, a well‑known selective serotonin reuptake inhibitor (SSRI), is widely used for the management of mental disorders and various types of chronic pain. Fluvoxamine blocks serotonin reuptake into the presynaptic nerve terminals, resulting in enhanced synaptic serotonin levels (Claassen et al. 1977; Fuller and Wong 1987). It is believed that attenuation of peripheral inflammation is also regarded as one of the possible mechanisms against depression, which has been employed for the treatment of major depressive disorders. For example, proinflammatory cytokines such as TNF‑α, IL‑6, and IL‑1β are implicated in the pathogenesis of depression (Dowlati et al. 2010). In particular, fluvoxamine has been noted with anti-inflammatory effect by regulating the function of ER-resident protein sigma-1 receptor (S1R), an important modulator for innate and adaptive immune responses (Rosen et al. 2019). Therefore, fluvoxamine manifested feasibility against septic shock, experimental autoimmune encephalomyelitis (EAE) (Ghareghani et al. 2017; Rosen et al. 2019), and cytokine storm resulted from COVID-19 infection (Lenze et al. 2020). These findings promoted us to hypothesize that fluvoxamine could be a good candidate drug for T1D prevention and treatment. To address this feasibility, we conducted studies in NOD mice, a well-established mouse model for human T1D. Our results revealed that fluvoxamine protected NOD mice from spontaneous T1D as manifested by the attenuated insulitis along with improved reservation of β mass. Mechanistically, fluvoxamine repressed PI3K-AKT signaling in CD4^+^ Teff cells to impair their glycolytic metabolism. As a result, the differentiation of Th1 and Th17 cells was significantly abrogated. These data support that administration of fluvoxamine could be a viable therapy against T1D in clinical settings.

## Results

### Administration of fluvoxamine prevents diabetes in NOD mice

**Fig. 1:**
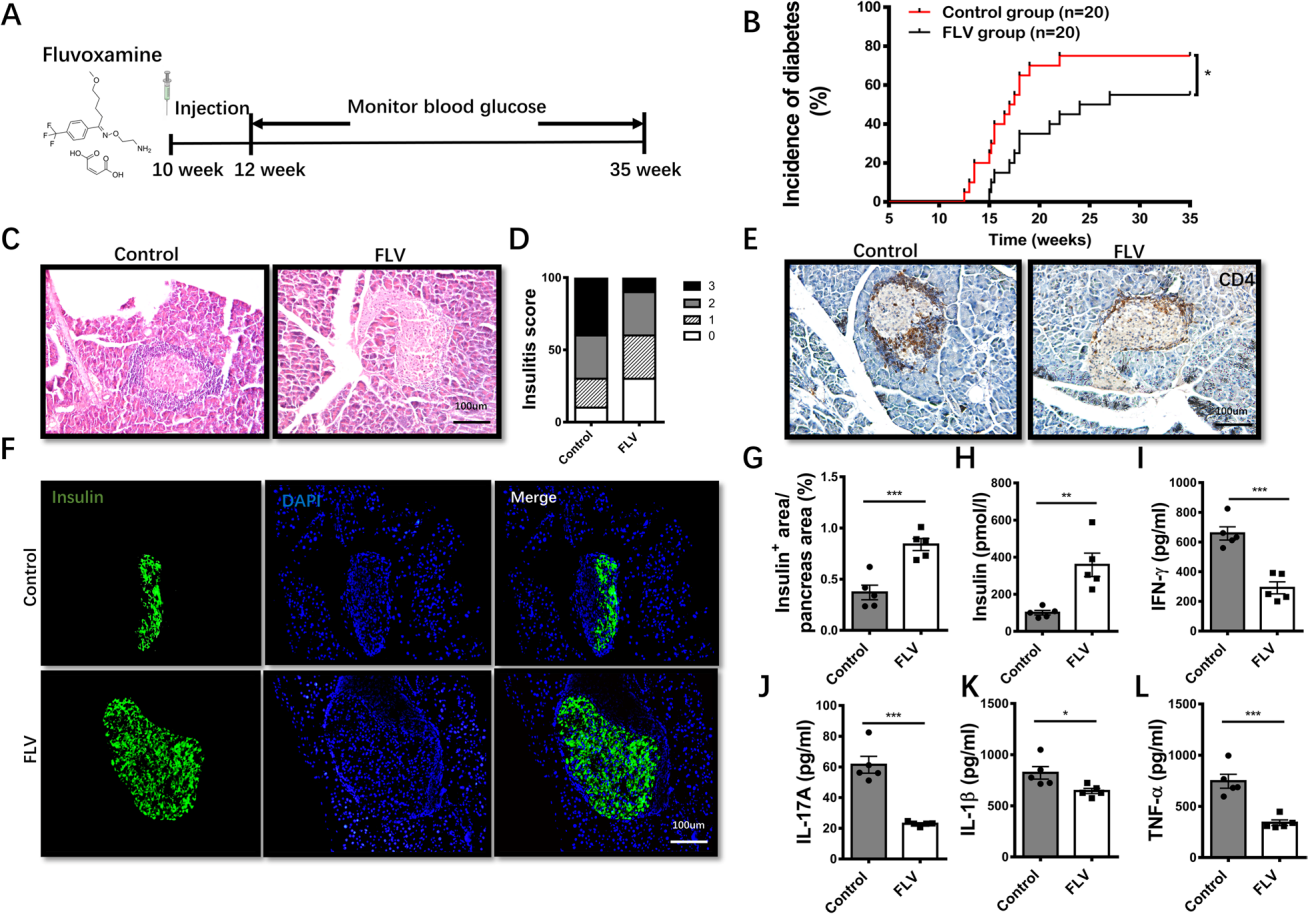
Administration of fluvoxamine attenuates T1D progression in NOD mice and preserves pancreatic insulin production. **A** Schematic diagram for in vivo experiments to determine the effect of fluvoxamine on T1D in NOD mice.** B** The incidence of diabetes in fluvoxamine-treated and vehicle-treated NOD mice (n = 20/group). **C** Insulitis was examined and **D** Insulitis scores in 12-week-old prediabetic fluvoxamine- and vehicle-treated NOD mice. **E** Immunohistochemical staining of CD4 in pancreatic sections of NOD mice at 12-week-old age. **F** Representative results of insulin immunostaining in the pancreas and **G** quantification of insulin positive areas in mice after fluvoxamine treatment or vehicle. The protein levels of **H** insulin, **I** IFN-γ, **J** IL-17A, **K** IL-1β and** L** TNF-α were detected by ELISA assays, respectively. Five mice per group were sacrificed at the same time point. Diabetes incidence was compared by log-rank test; differences in insulitis score was determined by χ^2^ test; in other figure parts statistical difference was analysed by unpaired Student’s *t* test. Data are expressed as mean ± SEM. *p < 0.05, **p < 0.01, ***p < 0.001, and ns, not significant

To address the effect of fluvoxamine on T1D development, 10-week-old female NOD mice were treated with either fluvoxamine (20 mg/kg) or the same amount of vehicle every other day for two weeks, and then monitored for the development of spontaneous T1D up to 35-week-old (Fig. 1A). As expected, administration of fluvoxamine substantially reduced diabetes incidence and delayed diabetes onset (Fig. 1B). The severity of insulitis was also reduced in fluvoxamine-treated mice relative to the control mice (Fig. 1C, D). Decreased islet infiltration was further confirmed by CD4 staining (Fig. 1E). Moreover, more structured islets and insulin-positive cells were noted in fluvoxamine-treated mice, while control mice manifested numerous shrunk islets along with a significant reduction of functional β cells (Fig. 1F, G), which were consistent with an increase of serum insulin levels in fluvoxamine-treated mice (Fig. 1H). In line with these observations, fluvoxamine administration remarkably decreased serum levels of proinflammatory cytokines IFN-γ, IL-17A, IL-1β, and TNF-α (Fig. 1I–L).

Other than T1D, NOD mice spontaneously develop autoimmune comorbidities, we therefore, assessed the impact of fluvoxamine on the systemic inflammation and autoimmunity in 12-week-old mice. Fluvoxamine-treated mice exhibited decreased infiltration of lymphocytes in the salivary gland, a hallmark prior to the development of Sjogren’s syndrome, but no obvious inflammation was observed in the colon, kidney, lung, and liver (Additional file 1: Fig. S1A). Fluvoxamine has been suggested with feasibility to induce agitation/anxiety (Omori et al. 2010), while our fluvoxamine-treated mice did not exhibit reduced interest in exploring the central region and locomotion in the open-field test (OFT) (Additional file 1: Fig. S1B-D), indicating that the dose of fluvoxamine employed in the study was not high enough to induce anxiety. Altogether, these results indicate that fluvoxamine possesses the potential to prevent T1D onset and insulitis progression in NOD mice.

### Fluvoxamine selectively reduces the number of CD4^+^ effector T cells

**Fig. 2:**
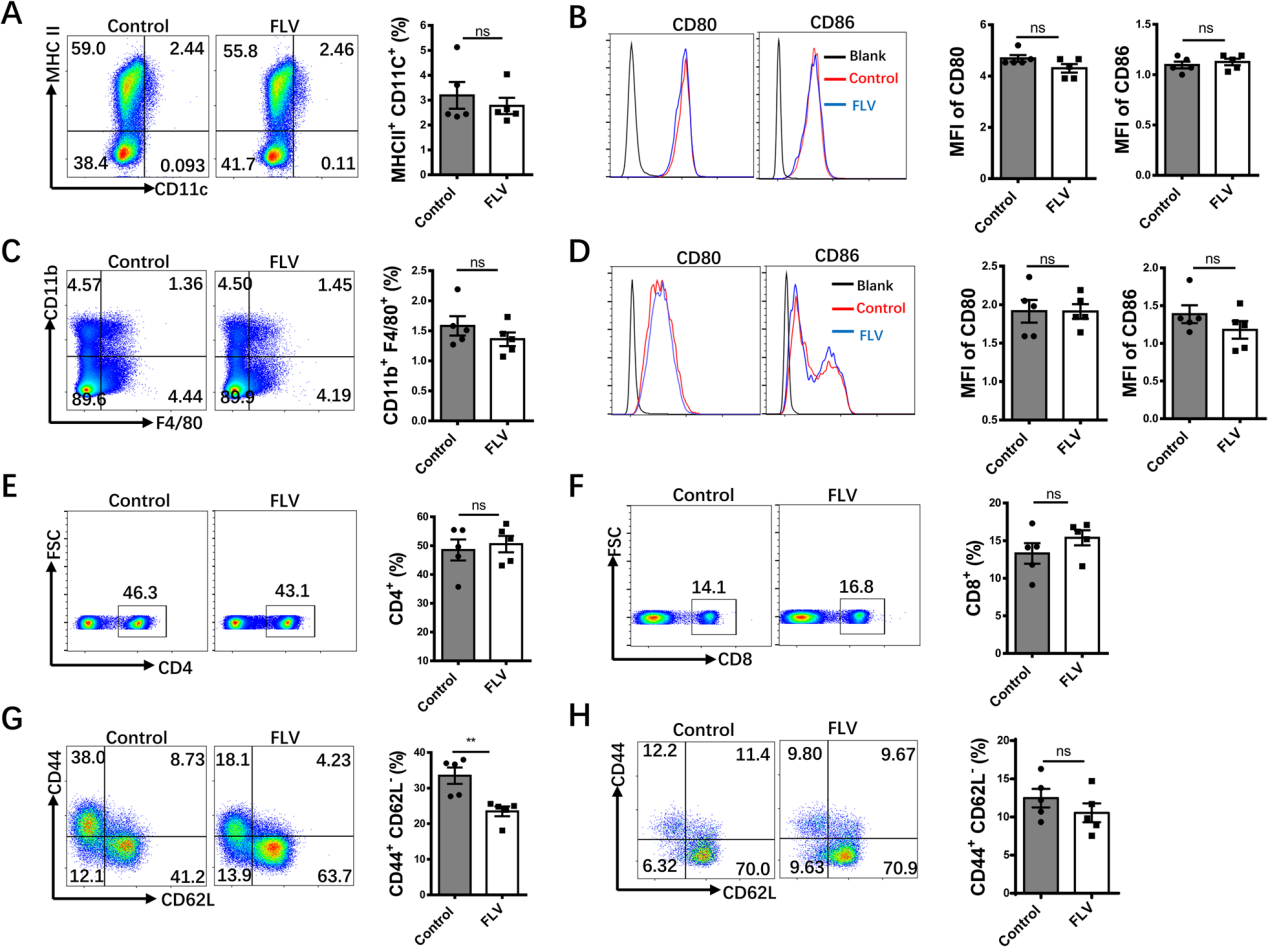
Fluvoxamine inhibits the activation of CD4^+^ T cells but does not affect dendritic cells, macrophages, and CD8^+^ T cells in the pancreatic lymph nodes (PLNs). PLN cells from 12-week-old fluvoxamine-treated and vehicle-treated mice were harvested and subject to flow cytometry analysis. Representative FACS plots of **A** DCs (MHCII^+^CD11C^+^) and **B** analysis of the MFI of CD80 and CD86. Representative FACS plots of **C** macrophages (CD11b^+^F4/80^+^) and **D** analysis of the MFI of CD80 and CD86. The frequencies of **E** CD4^+^, **F** CD8^+^, **G** CD4^+^CD44^high^CD62L^lo^ (CD4 effector T cells) and **H** CD8^+^CD44^high^CD62L^lo^ (CD8 effector T cells) are shown (n = 5 per group). Data are expressed as mean ± SEM. Statistical significance was calculated by unpaired Student’s *t* test. *p < 0.05, **p < 0.01, ***p < 0.001, and ns, not significant

To elucidate the cellular mechanisms underlying the protective effect of fluvoxamine observed above, we compared the differences of immune cells between 12-week-old control and fluvoxamine-treated mice. We first embarked on dendritic cells (DCs) and macrophages, as they play an essential role in autoimmune initiation and progression during the course of T1D development (Li et al. 2021). Unexpectedly, we failed to detect a perceptible difference either for the number of DCs or the expression of costimulatory molecules (CD80 and CD86) in the pancreatic lymph nodes (PLNs) of fluvoxamine-treated mice (Fig. 2A, B). Similar results were also observed for the proportion of macrophages (Fig. 2C) and the expression of costimulatory molecules (Fig. 2D). These observations prompted us to check T cell profiling in the PLNs. Although we failed to detect an obvious difference for the proportion of CD4^+^ (Fig. 2E) and CD8^+^ T (Fig. 2F) cells, the fluvoxamine-treated mice, however, exhibited a significantly lower proportion of CD44^hi^CD62L^lo^ CD4^+^ effector T cells (Fig. 2G) but not in CD8^+^ effector T cells (Fig. 2H). Collectively, these data suggest that fluvoxamine may selectively decrease the number of CD4^+^ effector T cells.

### Fluvoxamine preferentially inhibits the production of Th1 and Th17 subsets

**Fig. 3:**
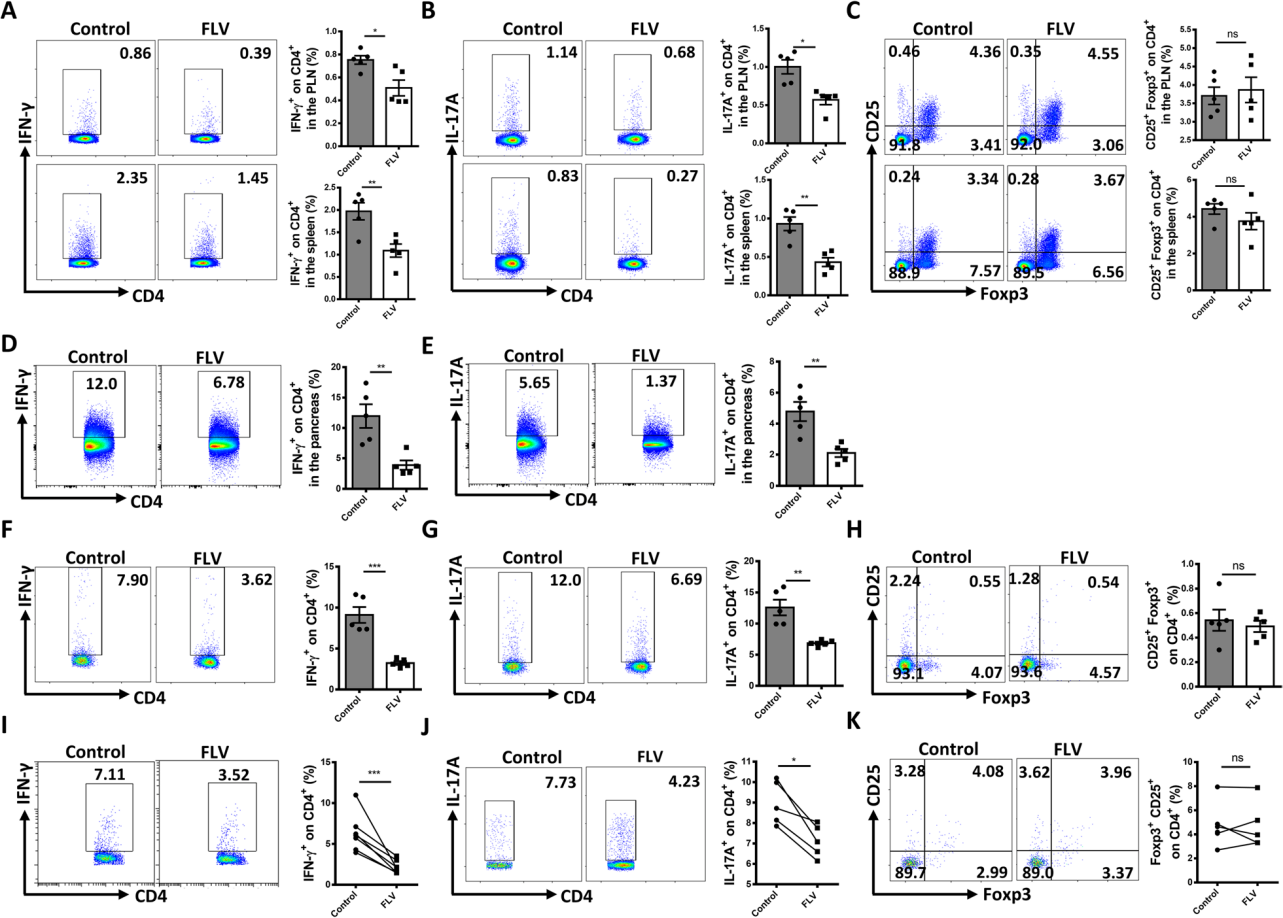
Fluvoxamine decreased the population of Th1 and Th17 cells in the PLNs, spleen, pancreas and peripheral blood. **A–C** PLN and splenic cells from 12-week-old fluvoxamine-treated and vehicle-treated mice were harvested and subject to flow cytometry analysis. Frequencies of** A** CD4^+^IFN-γ^+^ (Th1), **B** CD4^+^IL-17A^+^ (Th17), and **C** CD4^+^CD25^+^Foxp3^+^ (Treg) subsets were examined. **D–F** Blood from the mouse tail vein were collected and subjected to flow cytometry analysis. Frequencies of **D** Th1, **E** Th17, and **F** Treg subsets are shown as representative dot plot graphs. **G, H** Flow cytometry analysis of pancreatic cells from 12-week-old fluvoxamine-treated and vehicle-treated mice. Representative flow cytometry plots and frequencies of **G** Th1 and **H** Th17 effector T cells (n = 5 per group). **I–K** CD4^+^ T cells from peripheral blood of newly onset diabetic T1D subjects were stimulated with fluvoxamine or vehicle for 48 h, and the ratio of subtypes in CD4^+^ T cells were measured by flow cytometry. The cell frequency of **I** Th1 and **J** Th17 as well as **K** Treg cells was determined. The frequency of T cell subsets was investigated in ten donors. Data are expressed as mean ± SEM. Statistical significance was calculated by unpaired Student’s* t* test. *p < 0.05, **p < 0.01, ***p < 0.001, and ns, not significant

We next focused on the impact of fluvoxamine on CD4^+^ T cell subsets in NOD mice. In consistent with the above observations, the frequencies of CD4^+^IFN-γ^+^ (Th1) (Fig. 3A) and CD4^+^IL-17^+^ (Th17) (Fig. 3B) cells in the spleen and PLNs were significantly reduced following fluvoxamine treatment, but without a perceptible change in CD4^+^CD25^+^Foxp3^+^ regulatory T (Treg) cells (Fig. 3C). We then checked Th1 and Th17 subpopulations in the pancreas, and found that both of them were lower in fluvoxamine-treated mice (Fig. 3D and E). Similarly, the percentages of Th1 (Fig. 3F) and Th17 cells (Fig. 3G) were significantly lower in mice treated with fluvoxamine as compared to those control mice in the peripheral blood, while no obvious difference was observed in terms of peripheral Treg cells (Fig. 3H).

To confirm the above observations in T1D patients, we isolated CD4^+^ T cells from peripheral blood mononuclear cells (PBMCs) of patients with newly onset of T1D. The isolated cells were then stimulated with anti-CD3/28 in the presence or absence of fluvoxamine (10 μM) for 48 h, followed by flow cytometry analysis. Indeed, fluvoxamine significantly repressed the number of Th1 (Fig. 3I) and Th17 cells (Fig. 3J), but without a perceptible impact on Treg cells (Fig. 3K), confirming that fluvoxamine may selectively suppress the production of CD4^+^ effector T cells.

### Fluvoxamine inhibits the differentiation of Th1 and Th17 cells

**Fig. 4:**
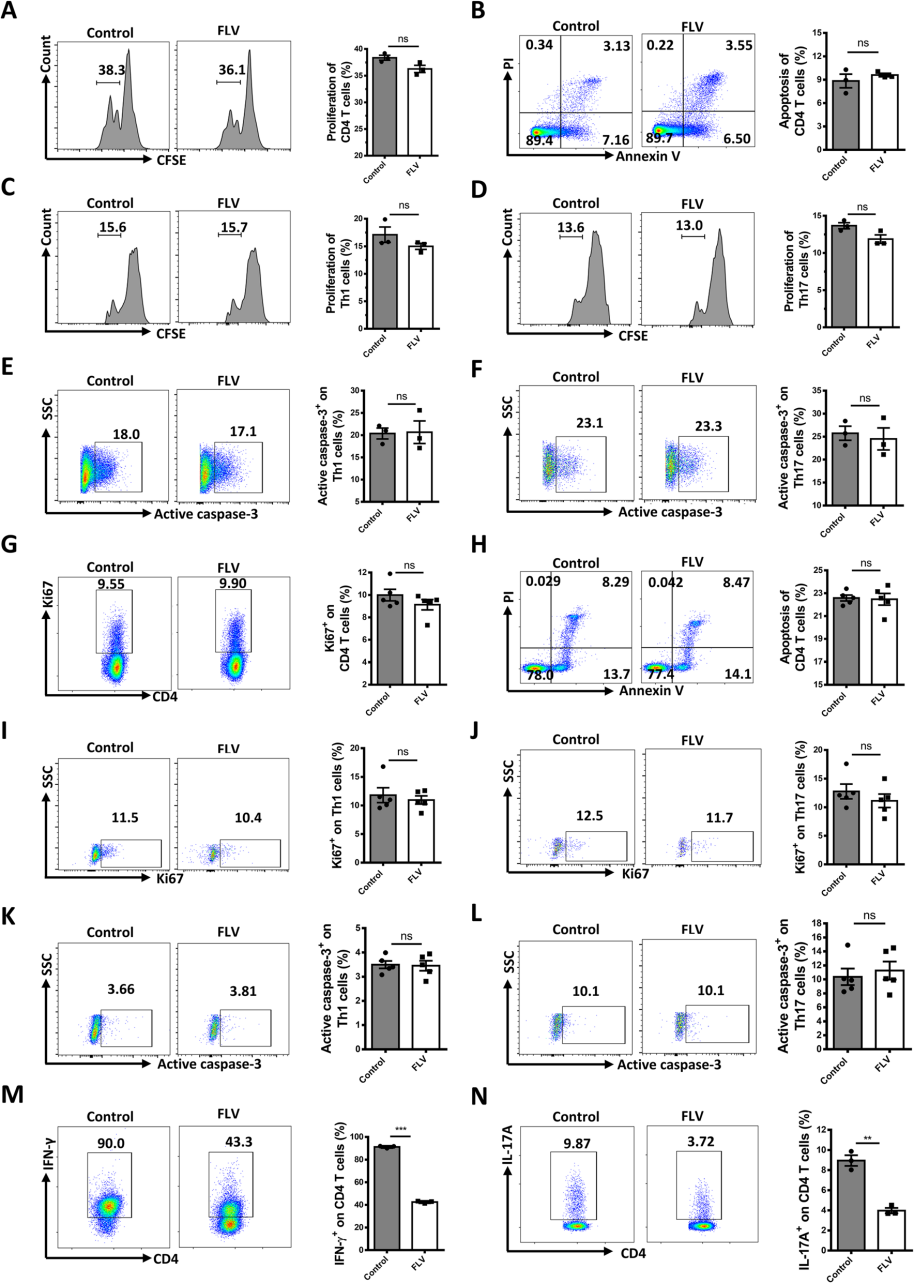
Fluvoxamine suppresses the differentiation of Th1 and Th17 cells rather than disturbing their proliferation and apoptosis. Splenic CD4^+^ T cells were isolated and stimulated with fluvoxamine (10 μM) or vehicle. The percentage of proliferated CD4^+^ T cells was defined by **A** Carboxyfluorescein Succinimidylester (CFSE) assay after 3 days of culture, and **B** apoptosis of T cells was determined by PI and Annexin V staining. Representative histograms of CFSE in **C** Th1 and **D** Th17 cells. Representative FACS plots and frequencies of active caspase-3 in **E** Th1 and **F** Th17 cells. Each dot represents the mean of three biological replicates. PLN cells from 12-week-old fluvoxamine- and vehicle-treated mice were harvested and subject to flow cytometry analysis. Representative FACS plots and frequencies of **G** CD4^+^Ki67^+^ T cells and **H** apoptotic CD4^+^ T cells determined by PI and Annexin V staining are shown. Percentage of Ki67^+^ proliferative **I** effector Th1 and **J** Th17 cells are shown by flow cytometry. **K** Apoptotic effector Th1 and **L** Th17 cells were indicated by staining active caspase-3 positive cells (n = 5 per group). Naïve CD4^+^ T cells purified from splenocytes were cultured under Th1 and Th17 conditions in vitro for 3 days in the presence of fluvoxamine or vehicle. **M** Th1 and **N** Th17 polarization efficiency was analyzed by flow cytometry. Each dot represents the mean of three biological replicates. Data are expressed as mean ± SEM. Statistical significance was calculated by unpaired Student’s *t* test. *p < 0.05, **p < 0.01, ***p < 0.001, and ns, not significant

To address the underlying basis of reduced Th1 and Th17 cells, we first checked the impact of fluvoxamine on CD4^+^ effector T cell proliferation and apoptosis. To this end, the isolated NOD splenic CD4^+^ T cells were stimulated with anti-CD3/28 in the presence or absence of fluvoxamine as above. Flow cytometry analysis of stimulated cells revealed comparable proliferation (Fig. 4A) and apoptosis (Fig. 4B) between two groups of cells. Given the relatively low proportions of Th1 and Th17 cells detected in total CD4^+^ T cells (Additional file 1: Fig. S2A and B), the proliferation and apoptosis of total CD4^+^ T cells may not accurately reflect the impact of fluvoxamine on Th1 and Th17 subsets. Therefore, we gated Th1 and Th17 cells for further analysis. Consistently, fluvoxamine treatment did not affect the proliferation and apoptosis of Th1 and Th17 cells, as evidenced by the comparable CFSE dilution (Fig. 4C and D) and active caspase-3 expression (Fig. 4E and F). To further confirm this result, we analyzed CD4^+^ effector (CD44^hi^CD62L^lo^) T cells from fluvoxamine-treated NOD mice, and similar results were obtained (Fig. 4G and H). Moreover, no perceptible difference was noted in terms of the percentages of Ki67^+^ and active caspase-3^+^ Th1/17 cells (Fig. 4I–L and Additional file 1: Fig. S2C and D) in vivo. These results support that the reduction of Th1 and Th17 cells in fluvoxamine-treated NOD mice is unlikely resulted from a proliferative or survival defect, which rendered us to examine whether fluvoxamine impacts CD4^+^ T cell differentiation. For this purpose, we isolated naïve CD4^+^ T cells from the spleen of female NOD mice, and then subjected to Th1, Th17 and Treg lineage commitment as described with fluvoxamine or vehicle. Addition of fluvoxamine significantly inhibited Th1 (Fig. 4M) and Th17 (Fig. 4N) differentiation, but no perceptible impact was noted on Treg polarization (Additional file 1: Fig. S3A). These data suggest that fluvoxamine represses the differentiation of naïve CD4^+^ T cells towards Th1 and Th17 cells, thereby protecting NOD mice from spontaneous type 1 diabetes.

### Fluvoxamine suppresses the secretion of inflammatory cytokines by CD4^+^ effector T cells

**Fig. 5:**
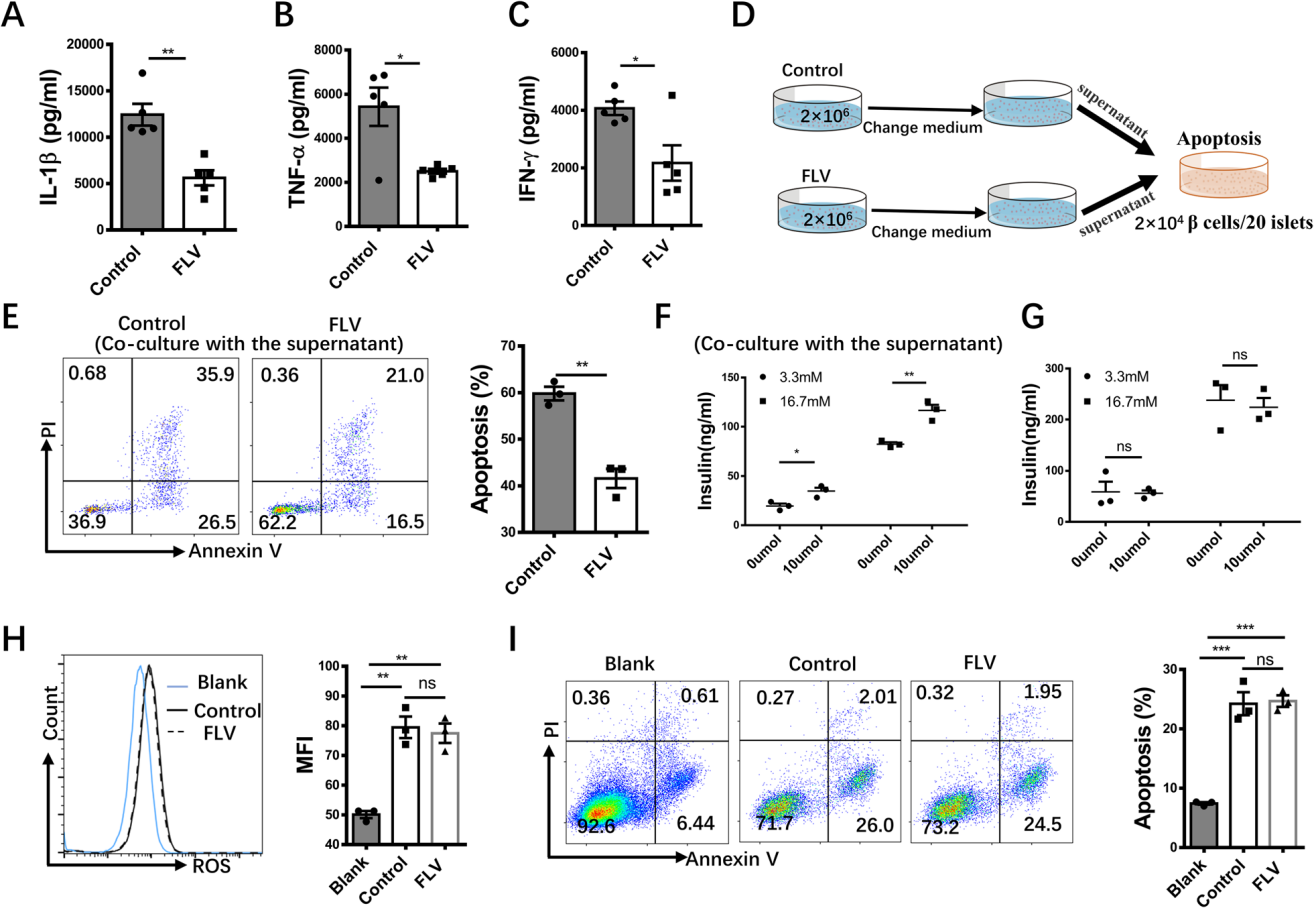
Fluvoxamine suppresses the secretion of inflammatory cytokines from CD4^+^ effector T cells. Splenic CD4^+^ T cells were isolated and pretreated with fluvoxamine or vehicle for 24 h, then changed medium and washed twice by complete medium, continued to culture the cells by new complete medium for 48 h. Supernatants were collected and **A** IL-1β, **B** TNF-α and** C** IFN-γ levels were analyzed by ELISA. **D** Schematic diagram of the co-culture system. Supernatants were co-cultured with NIT-1 cells for 24 h and then compared **E** apoptosis rate of NIT-1 cells in two groups. The isolated islets from NOD mice were randomly divided into four equals, following by adding culture supernatants of diabetogenic CD4^+^ T cells **F**, or treating with vehicle or fluvoxamine **G**. Then the islets were challenged with low (3.3 mM) or high (16.7 mM) glucose for 1 h, and insulin content in the supernatants was measured by ELISA. NIT-1 cells were treated with IL-1β, TNF-α and IFN-γ cytokines in the presence of fluvoxamine or vehicle for 24 h and tested the intracellular **H** accumulation of reactive oxygen species (ROS) and **I** apoptosis rate. The blank group consisted cells exclusively present in the medium. Each dot represents the mean of three biological replicates. Data are expressed as mean ± SEM. Statistical significance was calculated by unpaired Student’s *t* test. *p < 0.05, **p < 0.01, ***p < 0.001, and ns, not significant. Statistical difference in **H**, **I** was analyzed by one-way ANOVA

To further address whether fluvoxamine also regulates CD4^+^ effector T cell function, we examined its impact on inflammatory cytokine secretion from diabetologenic CD4^+^ T cells. For this purpose, diabetologenic CD4^+^ T cells were isolated from T1D newly onset NOD mice (diagnosed with T1D within one week), and then cultured in the presence or absence of fluvoxamine. The culture supernatants were collected and subjected to ELISA analysis of IL-1β, TNF-α and IFN-γ. Remarkably, fluvoxamine treatment significantly suppressed IL-1β, TNF-α and IFN-γ secretion from diabetogenic CD4^+^ T cells (Fig. 5A–C). To confirm this observation, the above culture supernatants were added into NIT-1 cell (a NOD-derived β cell line) or islet cultures, respectively (Fig. 5D). As expected, supernatants derived from fluvoxamine-treated diabetogenic T cells not only manifested significantly lower potency to induce NIT-1 cell apoptosis (Fig. 5E), but also less affected insulin secretion function of the islets (Fig. 5F).

To exclude the possibility that fluvoxamine directly impacts β cell function and viability, we first examined the impact of fluvoxamine on GSIS in islets isolated from NOD mice. No significant difference was observed in terms of GSIS between fluvoxamine-treated and control islets (Fig. 5G). We then treated NIT-1 cells with combination of IL-1β, TNF-α, and IFN-γ in the presence or absence of fluvoxamine, followed by analysis of reactive oxygen species (ROS) accumulation and apoptosis. Addition of fluvoxamine did not result in a significant change of intracellular ROS levels after cytokine stimulation (Fig. 5H), and no significant difference of apoptosis was observed between two groups of NIT-1 cells (Fig. 5I). Taken together, these results support that fluvoxamine not only suppresses Th1/Th17 polarization, but also attenuates their capability to secrete proinflammatory cytokines.

### Fluvoxamine inhibits glycolysis in CD4^+^ T cells

**Fig. 6:**
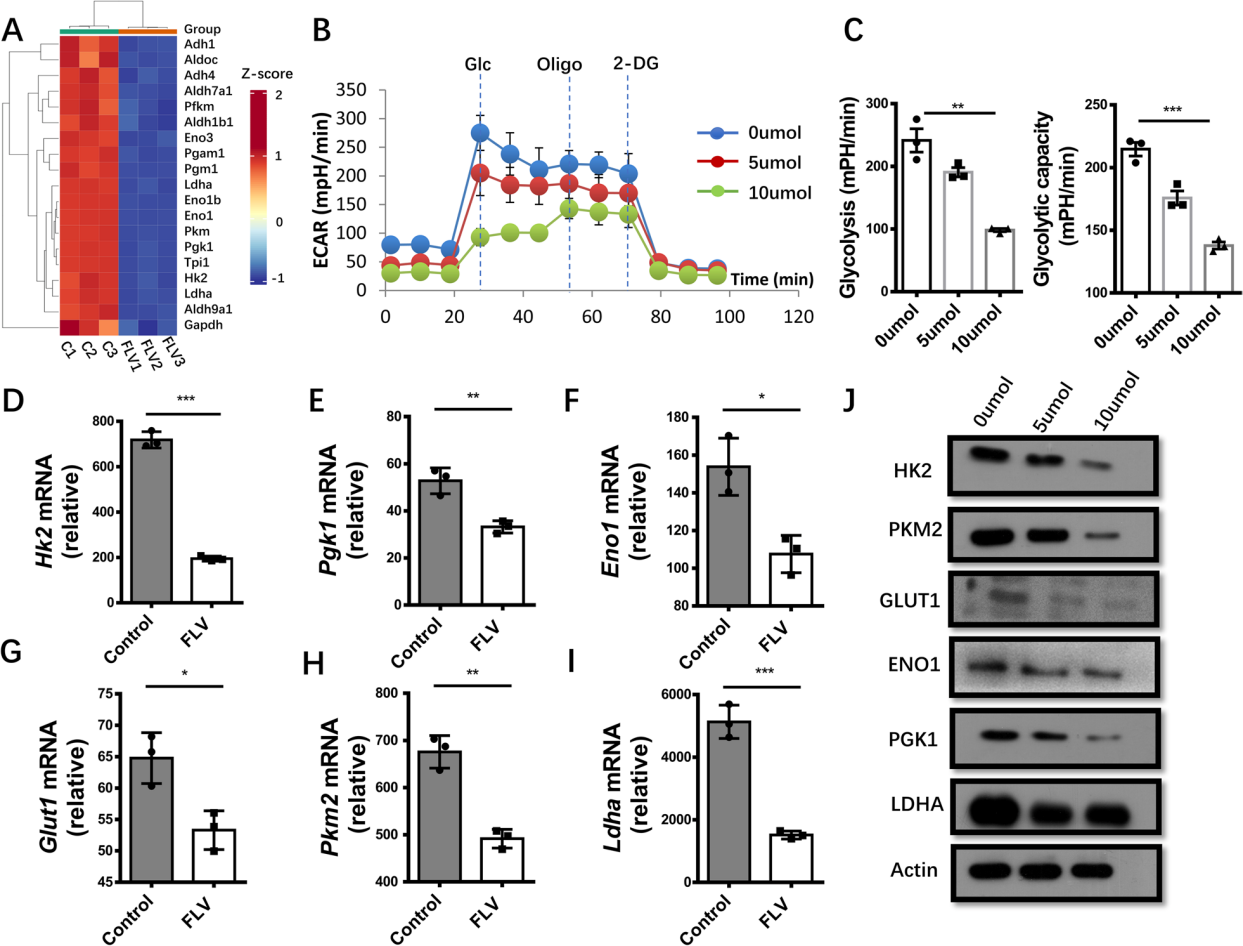
Fluvoxamine inhibits glycolysis of CD4^+^ T cells. **A** A heatmap shows the differentially expressed genes relevant to glycolysis between fluvoxamine- and vehicle-treated CD4^+^ T cells. The data were derived from 3 independent biological replicates. **B** CD4^+^ T cells were cultured with different concentration of fluvoxamine for 24 h in vitro and extracellular acidification rate (ECAR) was analyzed by an extracellular flux analyzer. **C** Glycolysis and glycolytic capacity in CD4^+^ T cells were determined with different dose of fluvoxamine treatment. **D–J** Real-time PCR analysis and Western blotting were performed to measure the expression of genes in the glycolysis pathway (*Hk2*, *Pgk1*, *Eno1*, *Glut1*, *Pkm2* and *Ldha*) in CD4^+^ T cells with/without fluvoxamine treatment. Each dot represents the mean of three biological replicates. Data are expressed as mean ± SEM. Statistical significance was calculated by unpaired Student’s *t* test. *p < 0.05, **p < 0.01, ***p < 0.001, and ns, not significant. Statistical difference in **C** was analyzed by one-way ANOVA

To gain insights into the molecular mechanisms by which fluvoxamine represses Th1 and Th17 polarization, we conducted deep RNA sequencing (RNA-seq) in fluvoxamine and vehicle-treated CD4^+^ T cells. Specifically, a set of signature genes involved in glycolytic metabolism were obviously downregulated in fluvoxamine-treated CD4^+^ T cells as shown in the heatmap (Fig. 6A). Given that glycolysis is essential for Th1 and Th17 cell differentiation, we, therefore, performed metabolic assays in fluvoxamine-treated CD4^+^ T cells. Fluvoxamine dose-dependently attenuated glycolytic process in CD4^+^ T cells as evidenced by the reduction of extracellular acidification rate (ECAR) (Fig. 6B). Particularly, the basal glycolytic rate was much lower than that of control cells coupled with an obvious reduction in the maximal glycolytic capacity following oligomycin induced mitochondria inhibition (Fig. 6C). Indeed, fluvoxamine-treated CD4^+^ T cells exhibited decreased mRNA levels for pivotal glycolytic genes, including *Hk2*, *Pgk1*, *Eno1*, *Glut1*, *Pkm2* and *Ldha* (Fig. 6D–I), which was further confirmed by Western blot analysis (Fig. 6J). Together, our results support that fluvoxamine disrupts glycolytic process, thereby attenuating Th1 and Th17 cell differentiation.

### Fluvoxamine represses PI3K/AKT signaling to attenuate glycolysis in CD4^+^ T cells

**Fig. 7:**
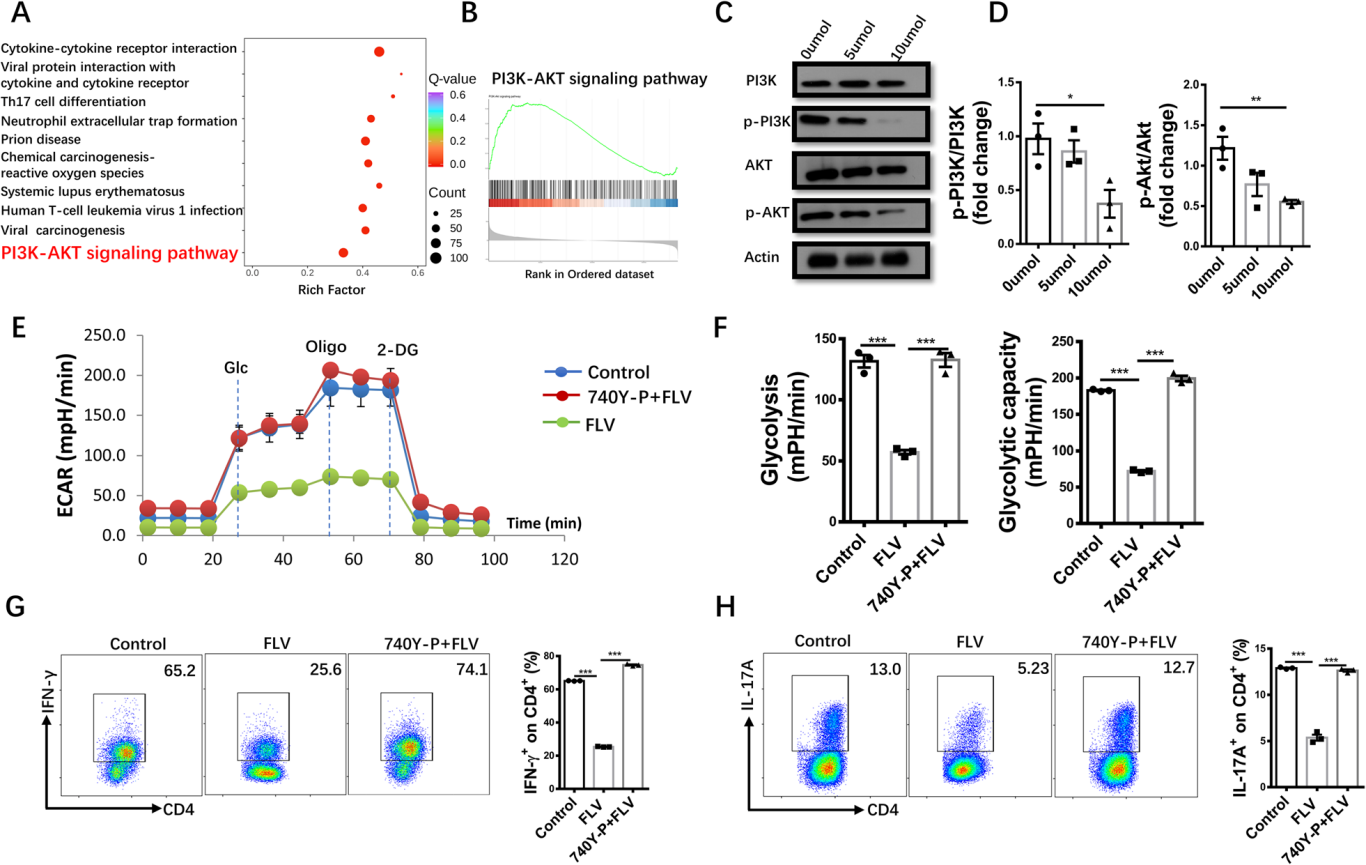
Fluvoxamine represses Th1 and Th17 differentiation and glycolysis by regulating the PI3K/AKT axis. **A** Genes downregulated in fluvoxamine-treated CD4^+^ T were subjected to KEGG pathway enrichment analysis. **B** Results for Gene Set Enrichment Analysis (GSEA) of PI3K-AKT signaling pathways. **C, D** The purified CD4^+^ T cells were cultured with fluvoxamine (0 μM, 5 μM, 10 μM) for 24 h. The protein expression of p-PI3K, p-AKT, PI3K and AKT was assessed. CD4^+^ T cells were cultured with fluvoxamine, vehicle or PI3K activator 740 Y-P for 24 h and **E** ECAR was analyzed by an extracellular flux analyzer. **F** Results for glycolysis and glycolytic capacity in CD4^+^ T cells with different treatment. **G** and **H** Naïve CD4^+^ T cells isolated from spleen were exposed to Th1-or Th17- inducing conditions under indicated culture conditions. **G** Th1 and **H** Th17 polarization efficiency were analyzed by flow cytometry. Each dot represents the mean of three biological replicates. Data are expressed as mean ± SEM. Significance was determined by one-way ANOVA, *p < 0.05, **p < 0.01, ***p < 0.001, and ns, not significant

Kyoto encyclopedia of genes and genomes (KEGG) pathway enrichment analysis of RNA-seq data indicated that fluvoxamine repressed PI3K/AKT signaling pathway (Fig. 7A). GSEA pre-ranked analysis further confirmed downregulated PI3K/AKT signaling in fluvoxamine-treated cells (Fig. 7B). We thus compared the expression changes of PI3K/AKT signaling between fluvoxamine-treated and control cells. Indeed, a dose-dependent reduction of p-PI3K and p-AKT levels was observed upon fluvoxamine treatment (Fig. 7C, D). To demonstrate that fluvoxamine indeed inhibits glycolysis and Th1/Th17 differentiation by regulating PI3K-AKT signaling, 740Y-P, a PI3K agonist, was added together with fluvoxamine into CD4^+^ T cell cultures. Remarkably, 740Y-P restored basal glycolytic rate and maximal glycolytic capacity in fluvoxamine-treated CD4^+^ T cells to a comparative level as that of control cells (Fig. 7E, F), indicating a crucial role for PI3K in fluvoxamine-induced metabolic reprogramming. Furthermore, addition of 740Y-P completely abolished the effect of fluvoxamine on Th1/Th17 program as evidenced by the comparative differentiation of Th1 and Th17 cells between fluvoxamine-treated and control CD4^+^ T cells (Fig. 7G, H). Overall, our results suggest that fluvoxamine represses PI3K/AKT signaling, by which it inhibits glycolysis to impair Th1/Th17 differentiation and their capacity to secrete inflammatory cytokines.

## Discussion

**Fig. 8:**
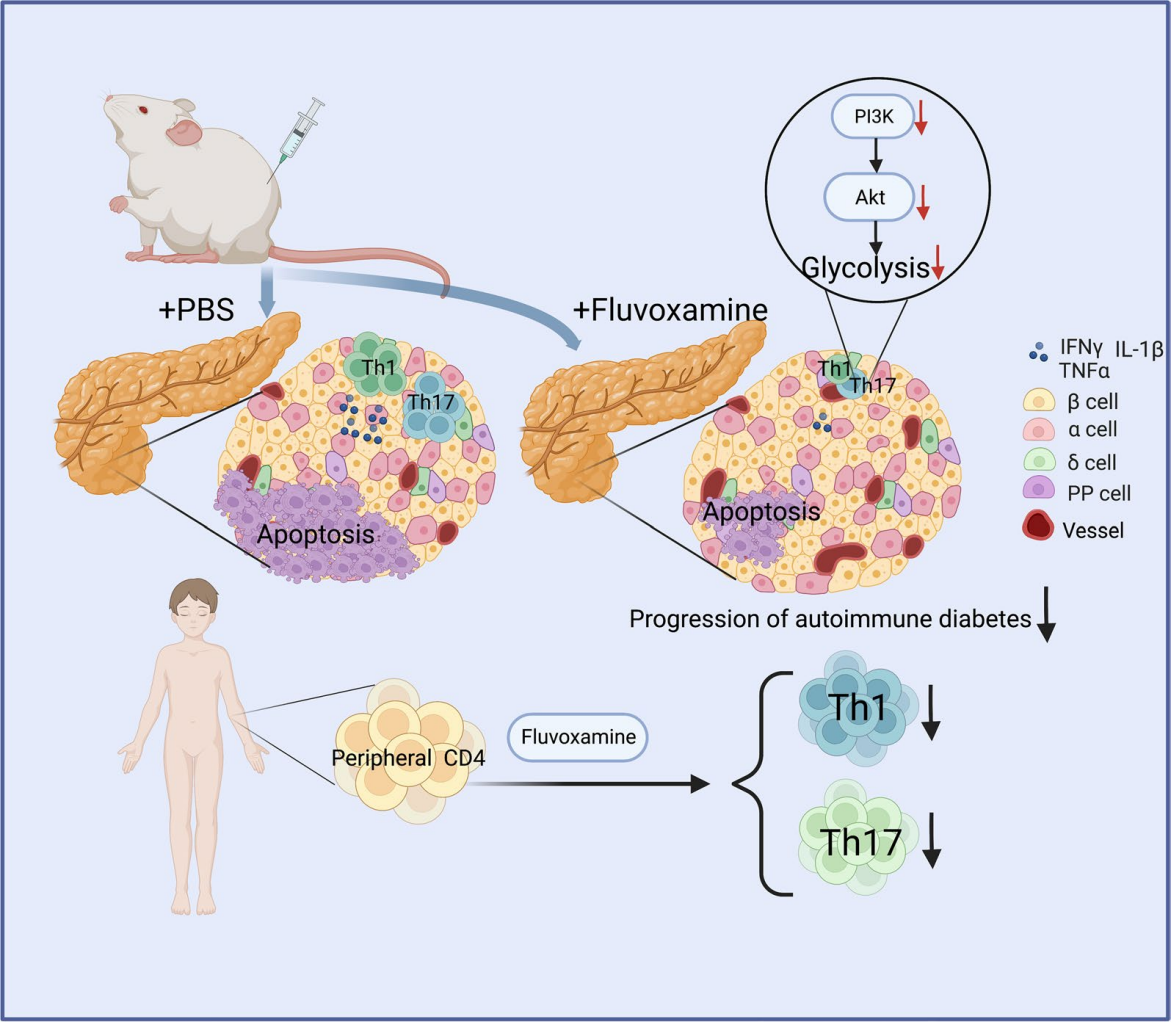
The scheme of fluvoxamine attenuates autoimmune progression in T1D setting. Fluvoxamine treatment inhibits glycolysis via restraining PI3K/AKT signaling. This metabolic change results in impaired differentiation and function of Th1 and Th17 cells. Consequently, administration of fluvoxamine delays diabetes onset and prevents autoimmune progression in T1D setting

Although fluvoxamine has been recognized with therapeutic potential for inflammatory and infectious diseases in clinical settings (Ghareghani et al. 2017; Rosen et al. 2019; Sukhatme et al. 2021), its effect on autoimmune disease, especially on T1D, however, has yet to be elucidated. In the present study, we treated 10-week-old NOD mice (a stage the mice already manifested autoimmune responses against pancreatic β cells) with fluvoxamine, and diabetes incidence was monitored until 35 weeks of age. We demonstrated convincing evidence supporting that administration of fluvoxamine provided protection for NOD mice against spontaneous T1D as manifested by the lower diabetes incidence, attenuated insulitis and well-preserved β cell function. Our data suggest that fluvoxamine could be a feasible drug against T1D in clinical settings (Fig. 8).

To elucidate the cellular foundation underlying the protective effects of fluvoxamine, we examined the impact of fluvoxamine on major types of immune cells that involved in T1D pathogenesis. Although the proportions and activation state of DCs, macrophages and CD8^+^ T cells were comparable between two groups, the fluvoxamine-treated mice manifested a significantly lower proportion of effector cells in total CD4^+^ T cells, as well as greatly reduced percentages of Th1 and Th17 cells in the PLNs, spleen, pancreas and peripheral blood. To confirm these results in humans, we isolated CD4^+^ T cells from T1D patients and stimulated with anti-CD3/28 in the presence or absence of fluvoxamine, and similar results were obtained. Given that fluvoxamine only manifested very limited impact on proliferation and apoptosis, we then examined its effect on T cell polarization. It was interestingly noted that naïve CD4^+^ T cells manifested a significantly lower potency to polarize to Th1 and Th17 effector cells in the presence of fluvoxamine.

Although multiple types of immunocytes are involved in T1D development, CD4^+^ T cells are known as the major culprits given their significant effect on the amplification of autoimmune responses, which rendered them an ideal target for immunotherapy to prevent or attenuate disease progression. However, autoreactive T cells are largely refractory to conventional immunomodulatory and immunosuppressive strategies in T1D patients (Gottlieb et al. 2010; Orban et al. 2011; Sherry et al. 2011), and as a result, characterization of check point targets that control T cell responses to β cells is still a formidable challenge. Previous studies have revealed that proinflammatory cytokines can damage the viability and functionality of pancreatic β cells (Sauter et al. 2015; Wachlin et al. 2003). Here we found that although fluvoxamine did not affect the apoptosis of NIT-1 cells and the function of pancreatic islets directly, treatment with fluvoxamine led to a notably reduced secretion of inflammatory cytokines by diabetogenic CD4^+^ T cells, including IL-1β, TNF-α, and IFN-γ. Consequently, the culture supernatant of fluvoxamine-treated diabetogenic T cells not only manifested significantly lower potency to induce NIT-1 cell apoptosis, but also less affected insulin secretion function of the islets.

In general, naïve CD4^+^ T cells are defined as CD44^lo^CD62L^hi^ cells, while the activated effector T cells are classified as CD44^hi^CD62L^lo^ cells. Recent advances in immunometabolism have shown that cellular metabolism plays a fundamental role in shaping T cell activation, differentiation and function. In particular, Th1 and Th17 cells primarily rely on glycolysis to support their effector function, while Treg cells are predominantly dependent on oxidative phosphorylation to maintain their suppressive function (Patel and Powell 2017). Therefore, suppression of glycolysis would attenuate naïve CD4^+^ T cells differentiate into proinflammatory effector Th17 and Th1 cells (Liu et al. 2023). In line with this notion, fluvoxamine potently inhibited glycolytic process and repressed the expression of glycolytic genes at both mRNA and protein levels in CD4^+^ T cells, thereby highlighting its function in targeting T cell metabolism. It is worthy of note that we failed to detect a marked difference for the proportion of CD4^+^ T cells in total PLN T cells of mice following fluvoxamine treatment, but those mice exhibited a significantly lower proportion of CD44^hi^CD62L^lo^ effector T cells in total PLN CD4^+^ T cells. This finding is in fact consistent with the observation of attenuated effector CD4^+^ T cell infiltration in the pancreatic islets following fluvoxamine treatment, which was most likely caused by the reduced activation and subsequent migration and expansion of autoreactive CD4^+^ effector T cells, rather than by the change of total CD4^+^ T cells in the peripheral lymphoid organs.

Of note, our RNA-seq data revealed that PI3K-AKT signaling pathway was downregulated in fluvoxamine-treated CD4^+^ T cells. Given that PI3K-AKT signaling is critical for promoting the expression of genes to maintain sustained glycolysis (Wofford et al. 2008), we thus speculated that fluvoxamine inhibits glycolysis in CD4^+^ T cells by repressing PI3K-AKT signaling. Indeed, a dose-dependent reduction in p-PI3K and p-AKT levels was observed upon fluvoxamine treatment, and supplement of PI3K agonist almost completely rescued the alteration of glycolysis and Teff signature in fluvoxamine-treated cells, supporting that fluvoxamine attenuates Th1 and Th17 program predominantly by inhibiting glycolysis via the PI3K-AKT signaling.

Although SSRI treatment is intended to increase the extracellular level of serotonin in the brain, it also increases serotonin levels in the peripheral tissues and blood. It is, therefore, reasonable to assume that serotonin participates in the pathogenesis of autoimmune diseases, as drugs targeting at serotonin signaling were certified to be beneficial in mouse models and clinical trials of multiple sclerosis (MS) and inflammatory bowel disease (IBD) (Benson et al. 2013; Coates et al. 2017; Malinova et al. 2018). Serotonin not only acts directly on Th1 cells to reduce their production of IFN-γ in MS patients, but also reduces the production of IL-17 and IFN-γ from Th17 in PBMCs isolated from MS patients (Sacramento et al. 2018). Although evidence supporting the connection between 5-HT and immune cells in T1D setting is lacking, we cannot completely exclude the feasibility that serotonin signaling may also provide positive synergic effect after fluvoxamine treatment.

According to psychiatric assessments, autoimmune diseases are usually associated with considerable psychiatric problems, including depression and anxiety. Some studies reported more than three times higher prevalence of depression in MS patients (Patten et al. 2017), and the rate of major depressive disorder in patients with rheumatoid arthritis was two to three times higher than that in the general population (Covic et al. 2009). Research studied the use of antidepressant drugs in MS supported their beneficial effects regarding depression related symptoms (Faissner et al. 2017), and on the other hand, fluvoxamine attenuated neuroinflammation and EAE severity in mice following MS induction (Ghareghani et al. 2017). In fact, studies have also implicated an increased prevalence of depressive disorders comorbid with anxiety in patients with T1D (Buchberger et al. 2016; Muscatello et al. 2017). Therefore, patients coupled with T1D-associated depression might benefit much from both the antidepressant and immunomodulatory effects of fluvoxamine. However, population-based studies are needed to prove the clinical benefits.

## Conclusion

In conclusion, administration of fluvoxamine prevented T1D development via its inhibitory effect on glycolysis in Th1 and Th17 program. Our results provided crucial mechanistic insights into the therapeutic benefits of fluvoxamine, which holds the potential to be a promising candidate drug for the treatment of T1D and other autoimmune diseases in clinical settings.

## Methods and materials

### Animals

Female NOD/ShiLtJ mice (8- to 9-week-old) were purchased from the Animal Model Research Center of Nanjing University (Nanjing, China). All mice were housed in a specific pathogen-free animal facility at the Tongji Medical College on a 12/12 h light/dark cycle. The Mice were housed in our animal facility for 1-week before the treatment of vehicle or fluvoxamine (20 mg/kg) (HY-B0103A, Medchem Express, Shanghai, China) by peritoneal injection every other day for two weeks. After the mice reached 12 weeks of age, non-fasting blood glucose was measured three times per week using an Accu-Check Advantage glucometer (Roche Diagnostics, Indianapolis, IN, USA) and classed as diabetic once two consecutive blood glucose exceeded 13.8 mmol/l. All experimental procedures were approved by the Tongji Hospital Animal Care and Use Committee in accordance with the National Institutes of Health (NIH) guidelines.

### Antibodies and reagents

Anti-mouse CD3e (100340), anti-mouse CD28 (102116), recombinant mouse IL-2 (575406), recombinant mouse IL-6 (575702), recombinant mouse TGF-β (763102), recombinant mouse IL-12 (577004), recombinant mouse IL-23 (589002), Brilliant Violet 421-conjugated anti-mouse CD4 (100443), PerCP anti-mouse CD8a (100731), PE-conjugated anti-mouse/human CD44 (103008), APC-conjugated anti-mouse CD62L (104412), PE/Cyanine7-conjugated anti-mouse IFN-γ (505826), Brilliant Violet 421-conjugated anti-mouse IL-17A (512321), PE-conjugated anti-mouse IL-17A (506904), Alexa Fluor^®^ 647 anti-mouse/rat/human FOXP3 (320014), FITC anti-mouse CD11c (117306), Brilliant Violet 421™ anti-mouse I-A/I-E (107620), PE/Cy7 anti-mouse CD86 (105014), and PE anti-mouse F4/80 (123110) were purchased from Biolegend (San Diego, CA, USA). Anti-phospho-PI3 Kinase p85 (Tyr458)/p55 (Tyr199) (4228S), anti-PI3 Kinase p85 (4292S), anti-phospho-AKT (Ser473) (D9E) XP^®^ Rabbit mAb (4060S), anti-AKT (9272S), anti-PKM2 (4053S) and anti-β-Actin (3700S) antibodies were obtained from Cell Signaling Technology (Danvers, MA, USA); anti-PGK1 (A12686), anti-ENO1 (A11448), and anti-LDHA (A1146) antibodies were got from Abclonal (Wuhan, China); anti-HK2 (22029-1-AP) and anti-Glut1 (21829-1-AP) antibodies were obtained from Proteintech (Wuhan, China); and 740 Y-P (HY-P0175) was ordered from Medchem Express (Shanghai, China).

### Cell purification and polarization

Naïve CD4^+^ T cells from 8- to 10-week-old NOD mice were enriched with a Naïve CD4^+^ T Cell Isolation Kit for mouse (130-104-453; Miltenyi Biotec, Auburn, CA, USA), and CD4^+^ T cells were enriched with a mouse CD4^+^ T Cell Isolation Kit (130-104-454; Miltenyi Biotec, Auburn, CA, USA) according to the manufacturer’s instruction. The cells were cultured in RPMI 1640 medium (plus β-mercaptoethanol) supplemented with 10% FBS, 1% GlutaMax, 1% sodium pyruvate, and 1% Pen/Strep (all from Gibco, Shanghai, China) for further studies. Naïve CD4^+^ T cells were activated with 10 μg/ml plate coated anti-CD3 and anti-CD28. Th1 cocktail (10 ng/ml IL-2 and 10 ng/ml IL-12), Th17 cocktail (10 ng/ml IL-1β, 10 ng/ml IL-6, 10 ng/ml IL-23, and 5 ng/ml TGF-β), and Treg cocktail (10 ng/ml IL-2 and 10 ng/ml TGF-β) were added to drive Th1, Th17 and Treg polarization, respectively.

### T cell proliferation assay

CD4^+^ T cells purified from the spleen of 8- to 10-week-old NOD mice were stained with Cell Tracer Carboxyfluorescein Succinimidylester (CFSE) (565082; Biolegend). The cells (1 × 10^6^ /ml) were plated in the anti-CD3 and anti-CD28 coated 96-well plate in triplicates, followed by FACS analysis after 3 days.

### Metabolic assays

In vitro differentiated cells were cultured in the presence of vehicle or fluvoxamine for 72 h, and washed extensively before the assay. ECAR was measured using a Seahorse XFe24 analyzer (Agilent Technologies, Santa Clara, CA, USA) according to the manufacturer’s instruction. In brief, the cells were seeded on XFe24 microplates that had been pre-coated with poly-D-lysine (Sigma) to immobilize cells. The basic assay media contained 2.5 μM dextrose and 2 mM glutamine. Injections of 2 μM oligmycin, 2 μM FCCP, 10 mM 2-deoxyglucose, and 0.5 μM rotenone/antimycin A were then performed sequentially. XFe Wave software (Agilent Technologies, Santa Clara, CA, USA) was used to analyze the data.

### RNA-sequencing and bioinformatic analysis

CD4^+^ T cells were stimulated with or without 10 μM fluvoxamine for 24 h and then collected. DESeq2 was used to analyze the differential expression between the two groups, and the *P* value was corrected using the Benjamini & Hochberg method. The corrected *P* value and |log2foldchange| are used as the threshold for significant difference of expression levels. The enrichment analysis was performed based on the hypergeometric test. For KEGG, the hypergeometric distribution test was performed with the unit of pathway. GO analysis was conducted based on the GO term. Construction of the cDNA and sequencing were performed in Wuhan Metware Biotechnology Co., Ltd (Wuhan, China).

### ELISA

Cytokine levels in the serum and culture supernatants, including TNF-α, IFN-γ, IL-1β, IL-4, IL-17A, TGF-β and IL-10, were measured using the commercial ELISA kits obtained from BD Biosciences (San Diego, CA, USA) and eBioscience (San Diego, CA, USA).

### GSIS assay

Pancreatic islets were isolated and GSIS was carried out using established methods (He et al. 2018). All islets obtained from 5- to 6-week-old NOD donors. Analysis of insulin concentration was carried out using an Ultrasensitive mouse insulin immunoassay kit (EZassay, China) following the manufacturer’s instruction. Serum insulin was determined using a Rat/Mouse Insulin ELISA kit (80-PINMS-E01, ALPCO, Salem, NH, USA) as instructed.

### Apoptosis assay

Cell apoptosis was measured by flow cytometry using an Annexin V-FITC/PI Apoptosis Detection Kit from Vazyme (A211-01; NanJing, China). Cells were collected and washed with cold PBS twice. The cells were then resuspended in 100 μl of Annexin V binding buffer and incubated with 10 μl FITC-conjugated Annexin V and 5 μl propidium iodide for 15 min in the dark. All samples were examined using a Miltenyi flow cytometer.

### Real-time PCR and western blot analysis

Total RNA extraction and cDNA synthesis were performed using the established techniques (Hu et al. 2016). Protein extraction and Western blot analysis were performed as previously described (Chen et al. 2021). The primer sequences for examined genes are listed in Additional file 1: Table S1. The relative expression levels for each target gene were calculated using the 2^−ΔΔCt^ method. Samples were excluded from analyses if mRNA or protein was not detected.

### Human samples

Blood samples were collected in Tongji Hospital from participants with newly diagnosed type 1 diabetes. Subjects with active infection, neoplasia, or other comorbidities were excluded from our study. CD4^+^ T cells were isolated from PBMCs and cultured with or without 20 μM fluvoxamine for 48 h. Consent form was obtained from each study subject, and the study was approved by the Tongji Hospital Human Assurance Committee.

### Statistical analysis

Data were represented as mean ± SEM. All in vitro studies were performed at least three times. Statistical analyses of the data, as indicated in each figure legend, were conducted with the GraphPad Prism 5 software (GraphPad Software Inc., San Diego, CA). Two-tailed Student’s *t*-test was conducted to compare values between two groups. In all statistical cases, differences with *p* values < 0.05 was considered as statistically significant.

## Data and material availability

All data needed to evaluate the conclusions in the paper are present in the paper and/or the additional files. Additional data related to this paper may be requested from the authors.

### Supplementary Information

**Additional file 1.** Additional figures and table.

## Abbreviations

SSRIs: Selective serotonin reuptake inhibitors
FLV: Fluvoxamine
T1D: Type 1 diabetes
NOD: Non-obese diabetic
PLNs: Pancreatic lymph nodes
Teff: Effector T cell
DCs: Dendritic cells
Foxp3: Forkhead box P3
Th17: T helper 17
Th1: T helper 1
PBMC: Peripheral blood mononuclear cell
Tregs: Regulatory T cells
PI3K: Phosphatidylinositol 3-kinase
AKT: Protein kinase B
NO: Nitric oxide
OFT: Open-field test
IFN-γ: Interferon γ
TNF-α: Tumor necrosis factor α
IL: Interleukin
S1R: Sigma-1 receptor
GSIS: Glucose stimulated insulin secretion
ROS: Reactive oxygen species
ECAR: Extracellular acidification rate
