# Supplementary material for: Fluvoxamine inhibits Th1 and Th17 polarization and function by repressing glycolysis to attenuate autoimmune progression in type 1 diabetes
Source: Mol Med. 2024 Feb 5;30:23. doi: 10.1186/s10020-024-00791-1 (PMC10845844; doi:10.1186/s10020-024-00791-1)
Supplement: Supplementary file 1 — Additional file 1. Additional figures and table. [file 10020_2024_791_MOESM1_ESM.docx]

**Additional file Information**

**Fluvoxamine inhibits Th1 and Th17 polarization and function by repressing glycolysis to attenuate autoimmune progression in type 1 diabetes**

Yuan Zou^1,^*, Jing Zhang^1,^*, Fei Sun^1^, Qianqian Xu^1^, Longmin Chen^1,2^, Xi Luo^1^, Ting Wang^1^, Qing Zhou^1^, Shu Zhang^1^, Fei Xiong^1^, Wen Kong^3^, Ping Yang^1^, Qilin Yu^1,†^, Shiwei Liu^4,†^, and Cong-Yi Wang^1,4,†^

^1^Department of Respiratory and Critical Care Medicine, the Center for Biomedical Research, NHC Key Laboratory for Respiratory Diseases, Tongji Hospital, Tongji Medical College, Huazhong University of Sciences and Technology, Wuhan, China

^2^Department of Rheumatology and Immunology, the Central Hospital of Wuhan, Tongji Medical College, Huazhong University of Science and Technology, Wuhan 430000, China

^3^Department of Endocrinology, Wuhan Union Hospital, Tongji Medical College, Huazhong University of Science and Technology, Wuhan 430000, China

^4^Shanxi Bethune Hospital, Department of Endocrinology, Shanxi Academy of Medical Sciences，Tongji Shanxi Hospital, Third Hospital of Shanxi Medical University, Taiyuan 030000, China

**Additional Figures**


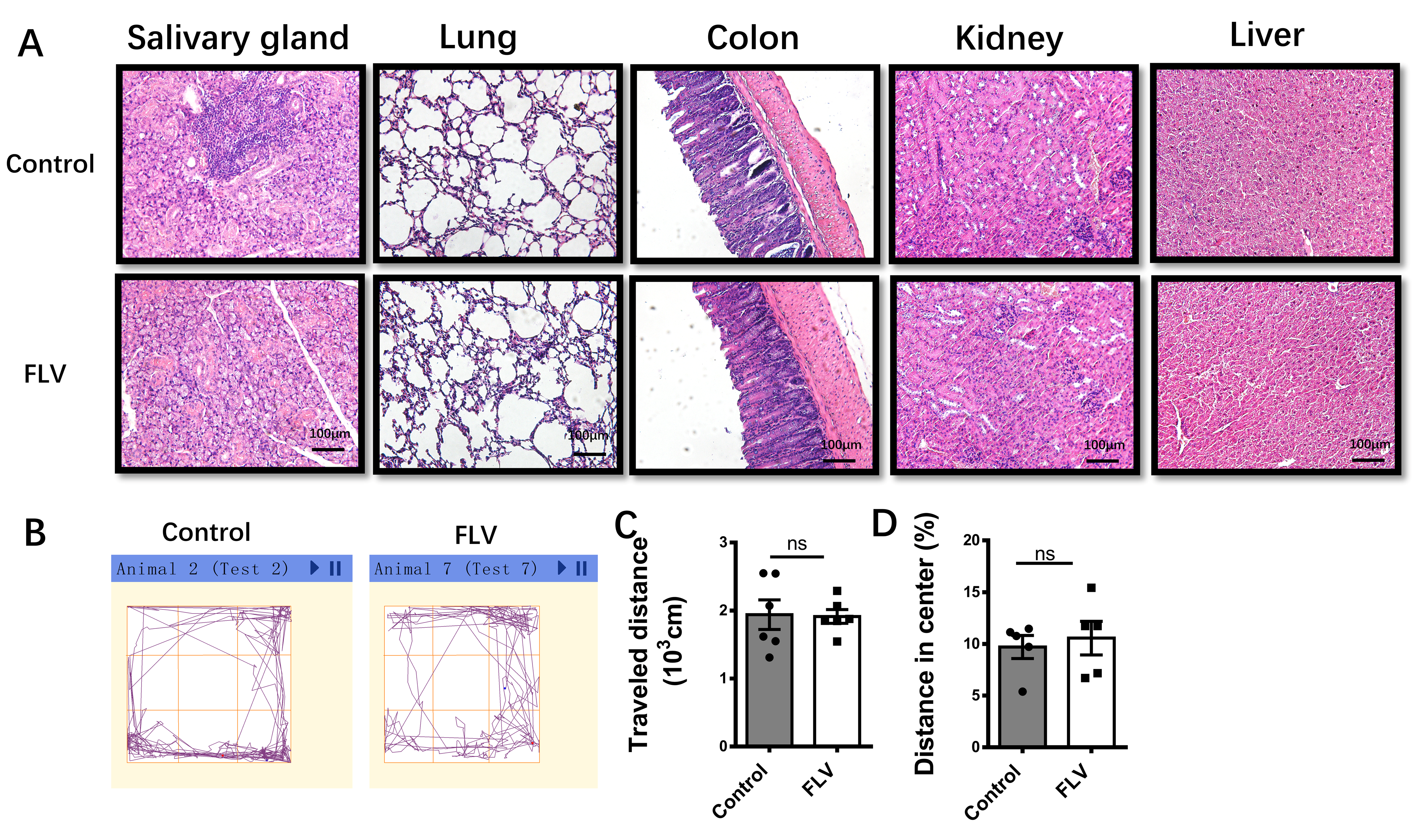


**Additional file 1: Fig. 1 Histological assessment of different tissues.**

**A** Representative picture of the salivary gland, lung, colon, kidney and liver of NOD mice at 12-week-old age. The images were taken under original magnification ×200. **B-D** Representative tracks of fluvoxamine-treated and PBS-treated mice in open-field test (OFT), as well as travel distance and percentage of distance in center area (n = 5). Data are expressed as mean ± SEM. Statistical significance was calculated by unpaired Student’s *t* test. ns, not significant.


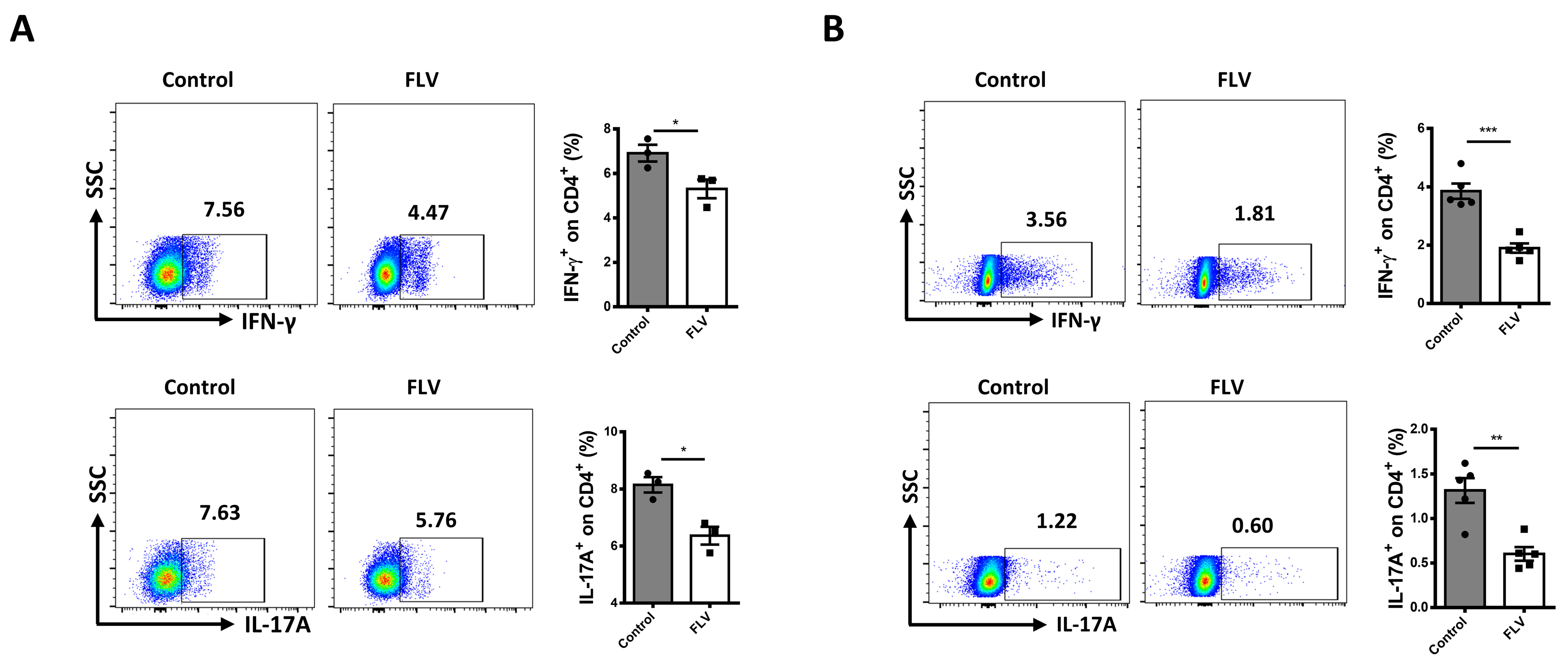


**Additional file 1: Fig. 2** **The percentages of Th1 or Th17 cells in total CD4^+^ T cells.**

**A** Splenic CD4^+^ T cells were isolated and stimulated with fluvoxamine (10μm) or vehicle. Th1 and Th17 cells in total CD4^+^ T were quantified and subjected to CFSE assay (Fig. 4C and D) and caspase-3 expression analysis (Fig. 4E and F). Each dot represents the mean of three biological replicates. **B** PLN cells from 12-week-old fluvoxamine- and vehicle-treated mice were harvested and used for flow cytometry analysis. Th1 and Th17 subsets are gated for subsequent proliferation (Fig. 4I and J) and apoptosis (Fig. 4K and L) analysis. n = 5 per group. Data are expressed as mean ± SEM. Statistical significance was calculated by unpaired Student’s *t* test. **p* < 0.05, ***p*< 0.01, ****p* < 0.001, and ns, not significant.


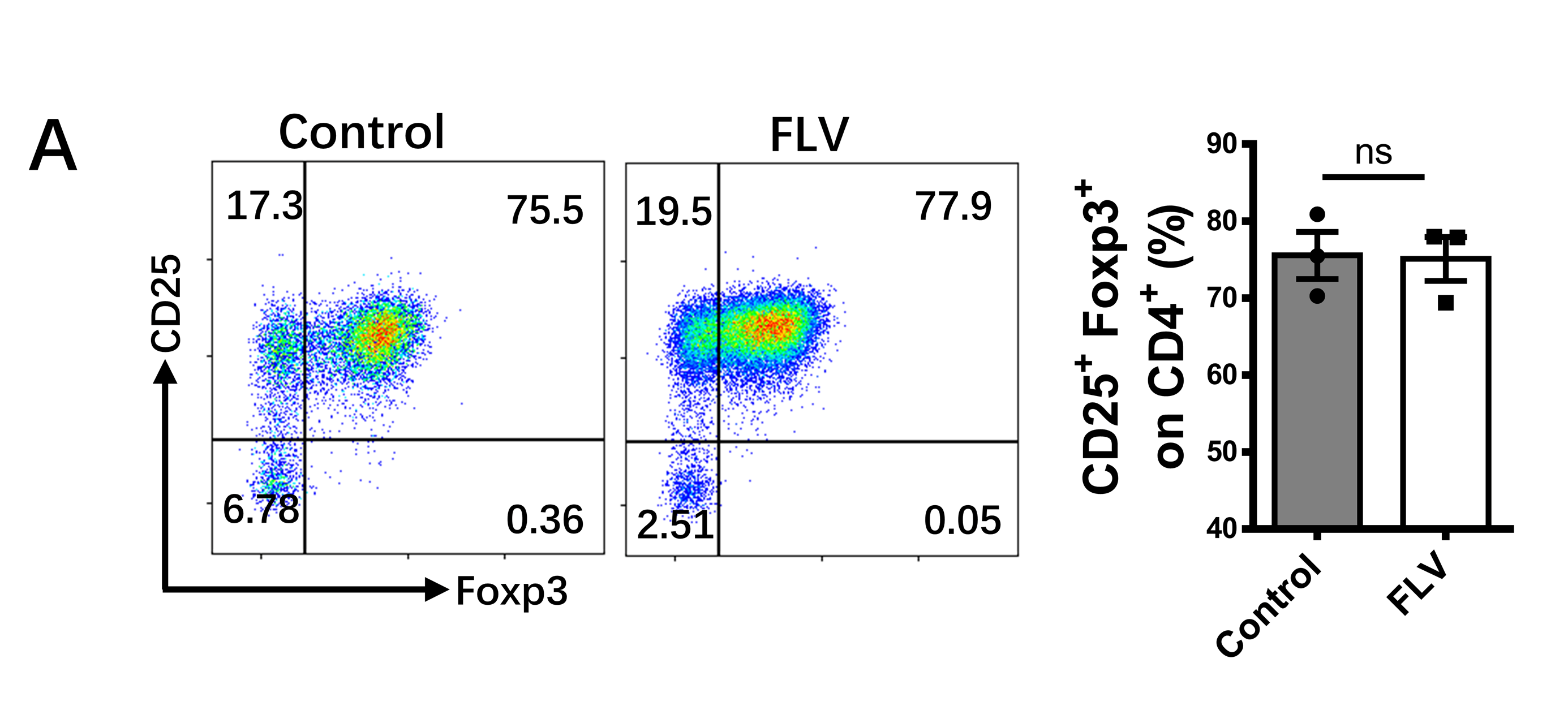


**Additional file 1: Fig. 3** **Fluvoxamine not impaired the differentiation of Tregs in vitro.**

Naïve CD4^+^ T cells purified from splenocytes were cultured under Treg conditions in vitro for 3 days in the presence of fluvoxamine or vehicle. **A** CD4^+^CD25^+^Foxp3^+^ (Treg) polarization efficiency was analyzed by flow cytometry. Each dot represents the mean of three biological replicates. Data are expressed as mean ± SEM. Statistical significance was calculated by unpaired Student’s *t* test. ns, not significant.

**Additional file 1: Table 1.** Primer sequences used in this study.

| Target gene | Forward sequence (5’-3’) | Reverse sequence (5’-3’) |
| --- | --- | --- |
| m-IL-17a | CAGACTACCTCAACCGTTCCAC | TCCAGCTTTCCCTCCGCATTGA |
| m-IFN-g | GATGCATTCATGAGTATTGCCAAG | GTGGACCACTCGGATGAGCTC |
| m-b-actin | CATTGCTGACAGGATGCAGAAGG | TGCTGGAAGGTGGACAGTGAGG |
| m-PKM2 | CGCCTGGACATTGACTCTG | GAAATTCAGCCGAGCCACATT |
| m-ENO1 | TGCGTCCACTGGCATCTAC | CAGAGCAGGCGCAATAGTTTTA |
| m-HK2 | CGACAGCATCATTGTGAAGG | TCCAGTCCACGGTTCTCTCT |
| m-GLUT1 | GCTTCCTGCTCATCAATCGT | CGACCCTCTTCTTTCATCTCC |
| m-PGK1 | GATTACCTTGCCTGTTGACTTTG | AGTGTCTCCACCACCTATGA |
| m-LDHA | ATTAAGCTGTCATGGGTGGGTC | CAGAGAGACACCAGCAACATTCA |
